# The Evaluation of Physiological Index Changes and Safety Work of Female Medical Staff With Different Medical Protection Standards in the Ward of COVID-19

**DOI:** 10.3389/fmed.2022.906140

**Published:** 2022-06-22

**Authors:** Min Zhao, Jianhui Zhao, Junbing Yan, Xiaoye Gao

**Affiliations:** ^1^Department of Internal Medicine, Fuzhou Pulmonary Hospital of Fujian, Fuzhou, China; ^2^Department of Epidemiology, School of Public Health, Southern Medical University, Guangzhou, China

**Keywords:** COVID-19, physiological indexes, working hours, security, personal protective equipment

## Abstract

**Background:**

Effective personal protective equipment (PPE) contribute to the prevention of COVID-19 infection. However, it is necessary to evaluate the potential risk of different medical protections in the isolation ward of COVID-19.

**Objectives:**

We aimed to explore the dynamics in physiological indexes of medical staff under primary and secondary PPE in the isolation ward of COVID-19 and provide the scientific basis for determining the safe work strategy.

**Materials and Methods:**

In this study, 30 female nurses were selected to simulate medical work under the primary or secondary PPE, respectively. The oral temperature, axillary temperature, heart rate, respiratory rate, blood oxygen saturation, and blood pressure were measured and recorded every 20 min. The subjective adverse symptoms were recorded every 30 min. The blood glucose and weight of the individuals were measured and recorded before and after the trial.

**Results:**

The results indicated that the median trial persistence time in the participants with moderate-intensity work wearing the secondary PPE (70.0 min) was much lower than that with moderate-intensity work wearing the primary PPE (180 min) and with light-intensity work wearing the primary PPE (110 min; *p* < 0.05). Importantly, the heart rate, oral/axillary temperature, and respiratory rate of physiological indexes of the participants under moderate-intensity work wearing the secondary PPE increased significantly faster than the primary PPE (*p* < 0.001), while blood oxygen saturation decreased significantly faster than the primary PPE (*p* < 0.001). In addition, the proportions of subjective adverse symptoms (such as dry mouth, dizziness, palpitations, and anhelation) were much higher than primary PPE (*p* < 0.001). The average sweat volume and blood glucose consumption of participants under moderate-intensity work wearing primary PPE were higher than secondary PPE (*p* < 0.001).

**Conclusion:**

The combination of an exacerbated workload and secondary PPE worn by COVID-19 healthcare workers increases the change in physiological indicators, and in some cases the adverse symptoms, which can affect and even suspend their medical work. For any medical institution, there is room for improvement in terms of bioethics of a “Job Well Done” to reduce the risks of medical activities under secondary PPE.

## Introduction

The coronavirus disease 2019 (COVID-19) has become a global pandemic since early 2020 (1), and it is currently in a state of normalized epidemic prevention and control in China. Severe acute respiratory syndrome coronavirus 2 (SARS-CoV-2) is a highly contagious virus that is mainly spread through close contact with infected people *via* respiratory droplets from coughing or sneezing. The isolation wards and medical staff of designated hospitals for COVID-19 are facing severe challenges. To ensure the health and safety of medical staff, the National Health Commission has successively issued the multi-edition of “*Technical Guidelines on Prevention and Control of COVID-19 in Medical Institutions*” (2–4). These files stipulated the types of personal protective equipment (PPE) and specifies the selection principles and wearing and taking off procedures of PPE for medical staff in different scenarios. Medical protective gowns, disposable medical caps, N95 medical masks, rubber surgical gloves, medical goggles medical shoe covers, and others are necessary PPE to prevent medical staff from being infected by the virus (5).

Many studies and experts have reported that the use of proper PPE for the medical staff can reduce the risk of infection for medical staff and patients (6, 7). However, when medical staff conduct their work with the necessary PPE, these PPE directly affect the physiology of medical staff, since it generates significant metabolic fatigue (8, 9). More importantly, this metabolic fatigue can not only impact operational capacity (10), but also increase the risk of accidents with PPE, increase cross-contamination, and contribute to physiological stress (9). N95 masks, as an important PPE, have good filtration efficiency for small particles, but also hinder airflow. In addition, the long-term use of N95 masks may cause subjective uncomfortable symptoms (11). Generally, the temperature of the micro-environment of medical staff under PPE is affected by both the indoor environment and the body’s heat dissipation, resulting in a series of changes in physiological indicators, and even headaches, fainting, and syncope (12, 13). Previously, it was reported that female COVID-19 healthcare workers wearing PPE more often experienced excessive sweating, fatigue, headache, shortness of breath, and dizziness during medical work (14). Furthermore, nurses work longer consecutive hours than other healthcare workers in COVID-19 isolation wards. Thus, medical staff in infectious disease hospitals are facing working stress, fatigue, and problems related to the use of PPE, especially nursing workers (15, 16).

The COVID-19 pandemic has heightened the use of PPE and hygiene activities among medical workers (17). Although the uses of proper PPE provides strong protection for medical staff in the ward of COVID-19, the potential damages for medical staff of PPE cannot be ignored during the COVID-19 pandemic. Currently, the initial observations from the United Kingdom, Italy, Singapore, and India reported that the PPE-induced heat strain among healthcare workers (18–20). However, the research on physiological indicators and safe working hours in medical staff under different standards of PPE has not been reported. Therefore, it is urgent to explore the medical staff body state in the different types of PPE, and further analyze the influence of medical PPE on the physiological indicators of medical staff, and provide the scientific basis for the establishment of medical safe working criteria and the improvement of medical PPE.

## Materials and Methods

### Study Participants

In this trial, 30 healthy female nurses aged 20–50 years with no underlying health problems were selected for performing light-/moderate-intensity medical work under the primary or secondary PPE. The normal ranges of heart rate, blood pressure, respiratory rate, oral temperature, axillary temperature, and blood oxygen are 60–100 beats/min, 90–139/60–89 mmHg, 12–24 beats/min, < 37.2 °C, < 37.0°C, and 95–100%. These participants were required to maintain a reasonable diet and adequate sleep before the initiation of the trial within 48 h, not take drugs, drink alcohol and coffee, not do vigorous exercise, and not eat food within half an hour before the initiation of the trial (21). Meanwhile, the baseline physiological indicators, such as heart rate, blood oxygen saturation, systolic blood pressure, diastolic blood pressure, respiratory rate, oral temperature, axillary temperature, and weight, were measured and made sure these indicators were within normal ranges (Supplementary Table 1). All participants were aware of the objectives, plans, and possible hazards of this trial, and signed the informed consent. The study was conducted in Fuzhou Pulmonary Hospital of Fujian, China, and was approved by the Ethical Committee of Fuzhou Pulmonary Hospital [2021-001(scientific research)-01].

### The Environment and Medical Personal Protective Equipment of This Trial

The trial was conducted in a simulated isolation ward in the Fuzhou Pulmonary Hospital, and a thermohygrometer was used to continuously detect the ambient temperature and humidity. During the trial, the indoor ambient temperature of the isolation ward ranged from 32 to 35°C, and the relative humidity was about 60%. The trials were carried out from 2 to 5 pm. This study used brisk walking (1.0 m/s) to simulate moderate-intensity activity and slow walking to simulate light-intensity activity (0.5 m/s) (22). Additionally, the determination of primary and secondary PPE was according to the technical guidelines and expert consensus on the prevention and control of COVID-19 (2–4, 23–26). In this study, the details of primary PPE and the secondary PPE are listed in Supplementary Table 2.

### Measuring Index and Instruments

We measured and recorded the heart rate, axillary/oral temperature, respiratory rate, and blood oxygen saturation of the research participant using corresponding instruments (Supplementary Tables 3 and 4) every 20 min before and during the trial. Additionally, the weight and blood glucose of all participants were measured and recorded before and after the trial. The respiratory rate was calculated by stopwatch and manual counting. All instruments have been regularly tested and qualified by the China National Institute of Metrology. The medical protective gowns and disposable isolation gowns used in the trial meet the national standard GB 19082-2009 (27, 28).

During the trial, the specially-assigned staff asked and recorded the subjective symptoms of the participants every 30 min. When the participants reported discomfort and inability to persist, the safety staff will stop the trial immediately and put them on medical observation. After the trial, the values of each physiological index and the duration of the individual study were measured and recorded immediately.

### Statistical Analysis

SPSS 24.0 and STATA/SE 15.0 (STATA Corp, College Station, TX, United States) were used for statistical analysis, and Graphpad Prism 8.0 was used for graph drawing. Continuous variables were presented as mean ± standard deviation (SD) or P_50_ (P_25_, P_75_) and tested by the *T*-test or Mann–Whitney test, whereas categorical variables were tested by chi-square tests or Fisher’s exact probability test. We modeled and compared the dynamics of heart rate, oral temperature, axillary temperature, respiratory rate, and blood oxygen saturation for the primary PPE and secondary PPE groups during the trial duration using generalized estimating equations (GEE). The value of *p* < 0.05 is considered statistically significant.

## Results

### Comparison of Percentage of Trial Terminated Between Participants Under Primary Personal Protective Equipment and Secondary Personal Protective Equipment

**FIGURE 1:**
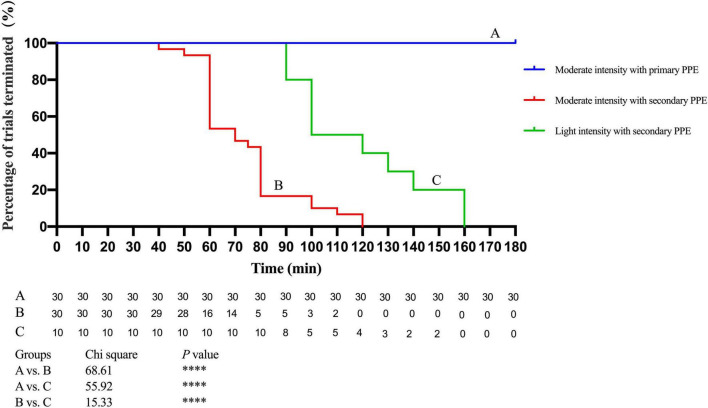
Analysis of trail duration of participants with secondary personal protective equipment (PPE) and moderate-intensity with secondary or primary PPE. ^*⁣*⁣**^p < 0.0001. The *p* values were calculated using the Log-rank test. PPE, personal protective equipment.

The results indicated that all 30 participants with moderate-intensity work wearing the primary PPE persisted for 180 min of the trial, but the shortest persistence time was only 40 min and the longest was 120 min for the participants with secondary PPE and none of them completed the 180 min whole trial (Figure 1). The proportion of trial terminated of participants under the primary PPE was far more than the secondary PPE (log-rank test, *p* < 0.01). In addition, the median time of participants with secondary PPE performing light-intensity work was 110 min (the range is 90–160 min), which was between moderate-intensity work with secondary PPE and moderate-intensity work with primary PPE (log-rank test, *p* < 0.01; Figure 1).

### Analysis of the Physiological Index Dynamic of Participants Under Primary Personal Protective Equipment and Secondary Personal Protective Equipment

**FIGURE 2:**
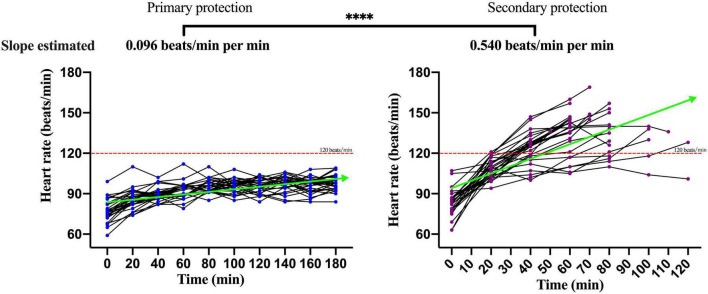
The dynamics of heart rate of participants wearing primary PPE and secondary PPE. ^*⁣*⁣**^p < 0.0001. The slopes of heart rate during the trial were calculated by generalized estimating equations. PPE, personal protective equipment.

**FIGURE 3:**
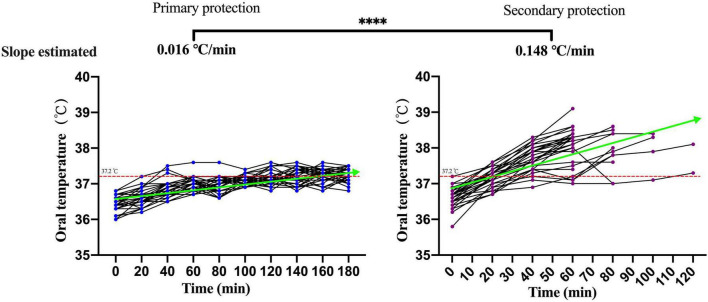
The dynamics of oral temperature of participants with primary and secondary PPE. ^*⁣*⁣**^p < 0.0001. The slopes of oral temperature during the trial were calculated by generalized estimating equations. PPE, personal protective equipment.

**FIGURE 4:**
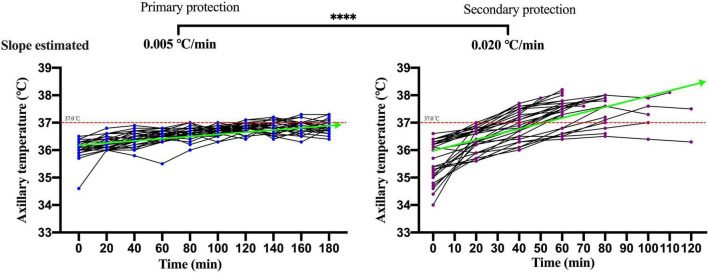
The dynamics of axillary temperature of participants with primary and secondary PPE. ^*⁣*⁣**^p < 0.0001. The slopes of axillary temperature during the trial were calculated by generalized estimating equations. PPE, personal protective equipment.

**FIGURE 5:**
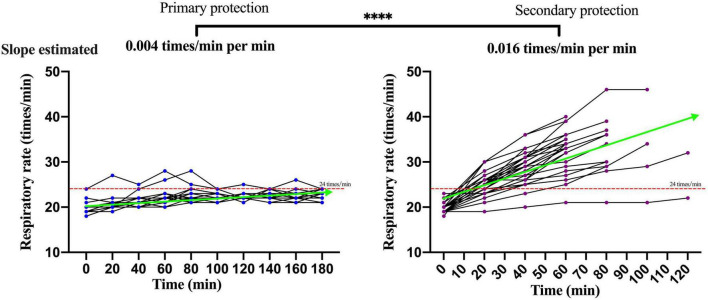
The dynamics of respiratory rate of participants with primary and secondary PPE. ^*⁣*⁣**^p < 0.0001. The slopes of respiratory rate during the trial were calculated by generalized estimating equations. PPE | personal protective equipment.

**FIGURE 6:**
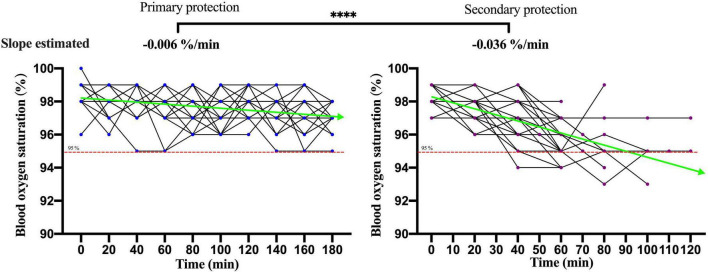
The dynamics of blood oxygen saturation of participants with primary and secondary PPE. ^*⁣*⁣**^p < 0.0001. The slopes of blood oxygen saturation during the trial were calculated by generalized estimating equations. PPE, personal protective equipment.

The changes of the physiological index in participants performing moderate-intensity work under secondary PPE were greater than primary PPE using the paired *T*-test or the Wilcoxon matched-pairs signed-rank test (Table 1). The change speeds of heart rate, oral temperature, axillary temperature, respiratory rate, and blood oxygen saturation were estimated using GEE. As shown in Figure 2, the change speeds of heart rate in participants performing moderate-intensity work wearing secondary PPE (0.54 beats/min) were faster than the primary PPE (0.0096 beats/min; *p* < 0.0001). Similarly, the ascending speeds of oral/axillary temperature of participants performing moderate-intensity work wearing secondary PPE (0.148 and 0.020°C/min) were also faster than the primary PPE (0.016 and 0.005°C/min; *p* < 0.0001; Figures 3, 4). Meanwhile, the respiratory speeds of participants with moderate-intensity work wearing primary PPE and secondary PPE were 0.016 and 0.004 beats/min, the difference was statistically significant (*p* < 0.0001; Figure 5). In addition, the results showed that the descent speed of blood oxygen saturation of participants with moderate-intensity work under secondary PPE was 0.036%/min, much faster than 0.006%/min of participants under the primary PPE (*p* < 0.0001; Figure 6). Additionally, the physiological index changes of participants with secondary PPE performing light-intensity work was between moderate-intensity work with secondary PPE and moderate-intensity work with primary PPE (*p* < 0.01; Table 2).

**TABLE 1:** The analysis of physical indicators of participants between primary personal protective equipment (PPE) and secondary PPE after trial.

Variable	Primary PPE (*n* = 30)	Secondary PPE (*n* = 30)	*P* value^a^
Heart rate, beats/min	98.67 ± 6.01	139.8 ± 12.75	< 0.001
Blood oxygen, %	97.00 (97.00, 98.00)	95.00 (95.00, 96.00)	< 0.001
Systolic blood pressure, mmHg	108.87 ± 7.59	105.43 ± 14.83	0.295
Diastolic blood pressure, mmHg	68.5 (65.00, 72.25)	64.50 (61.75, 72.50)	0.147
Respiratory rate, beats/min	23.00 (22.00, 23.00)	34.00 (33.00, 36.00)	< 0.001
Oral temperature, °C	37.30 (37.10, 37.40)	38.25 (37.98, 38.50)	< 0.001
Axillary temperature, °C	36.90 (36.78, 37.00)	37.70 (37.30, 37.90)	< 0.001
Blood glucose, mmol/L	5.36 ± 0.93	4.57 ± 0.98	0.003

*^a^p values were calculated using the paired T-test or the Wilcoxon matched-pairs signed-rank test.*

*PPE, personal protective equipment.*

**TABLE 2:** Comparison of the physiological index changes of participants between light-intensity with secondary PPE and moderate-intensity with secondary or primary PPE.

Variable	Group			*P* value^a^	*P* value^b^
	Light-intensity with secondary PPE	Moderate-intensity with secondary PPE	Moderate-intensity with primary PPE		
Heart rate, beats/min per min	0.228	0.540	0.096	0.033	< 0.0001
Systolic blood pressure, mmHg per min	–0.086	–0.070	0.021	0.272	< 0.0001
Diastolic blood pressure, mmHg per min	–0.04	–0.045	0.005	0.466	0.004
Respiratory rate, beats/min per min	0.057	0.148	0.016	0.012	< 0.0001
Oral temperature, °C per min	0.007	0.016	0.004	0.019	< 0.0001
Axillary temperature, °C per min	0.006	0.02	0.005	0.003	0.007
Blood oxygen saturation, % per min	–0.022	–0.036	–0.006	0.039	< 0.0001

*^a^The p value was calculated for light-intensity with secondary PPE vs. moderate-intensity with secondary PPE.*

*^b^The p value was calculated for light-intensity with secondary PPE vs. moderate-intensity with primary PPE.*

*PPE, personal protective equipment.*

### Analysis of the Subjective Adverse Symptoms of the Participants With Primary Personal Protective Equipment and Secondary Personal Protective Equipment During the Trial

The results indicated that the proportion of subjective symptoms, such as dry mouth, dizziness, palpitations, and anhelation of the participants, during the trial rose with the increase of the trial time under the primary PPE and the secondary PPE (*p* < 0.001; Supplementary Table 5). Furthermore, the proportions of subjective adverse symptoms, such as dry mouth, dizziness, palpitations, and anhelation after 30 and 60 min of the trial, in the participants with moderate-intensity work wearing the secondary PPE were higher than that of the participants wearing the primary PPE (*p* < 0.001; Table 3). Additionally, the proportions of subjective adverse symptoms, such as anhelation and palpitations after 30 and 60 min of the trial, in the participants with the secondary PPE performing light-intensity work were lower than moderate-intensity work but similar to the participants with the primary PPE performing moderate-intensity work (Table 3).

**TABLE 3:** Comparison of subjective symptoms of participants between light intensity with secondary PPE and moderate intensity with secondary or primary PPE after initiation of trial at 30 and 60 min.

Time		Group			*P* value^a^	*P* value^b^	*P* value^c^
	Symptoms	Light intensity with secondary PPE	Moderate intensity with secondary PPE	Moderate intensity with primary PPE			
30 min	Dry mouth	6 (60%)	24 (80.00%)	9 (30.00%)	0.399	0.187	< 0.001
	Anhelation	0 (0%)	19 (63.33%)	1 (3.33%)	0.002	> 0.999	< 0.001
	Dizziness	0 (0%)	7 (23.33%)	0 (0.00%)	0.23	−	< 0.001
	Palpitations	1 (10%)	24 (80.00%)	1 (3.33%)	< 0.0001	0.442	< 0.001
60 min	Dry mouth	7 (70%)	25 (83.33%)	18 (60.00%)	0.648	0.85	0.011
	Anhelation	0 (0%)	20 (66.67%)	1 (3.33%)	< 0.0001	> 0.999	< 0.001
	Dizziness	1 (10%)	25 (83.33%)	0 (0.00%)	< 0.0001	0.25	< 0.001
	Palpitations	2 (20%)	22 (73.33%)	1 (3.33%)	0.009	0.149	< 0.001

*^a^The p value was calculated for light intensity with secondary PPE vs. moderate intensity with secondary PPE.*

*^b^The p value was calculated for light intensity with secondary PPE vs. moderate intensity with primary PPE.*

*^c^The p value was calculated for moderate intensity with secondary PPE vs. moderate intensity with primary PPE.*

*PPE, personal protective equipment.*

### The Sweat Volume and Blood Glucose Consumption of Participants Before and After Trial Under Primary Personal Protective Equipment and Secondary Personal Protective Equipment

**FIGURE 7:**
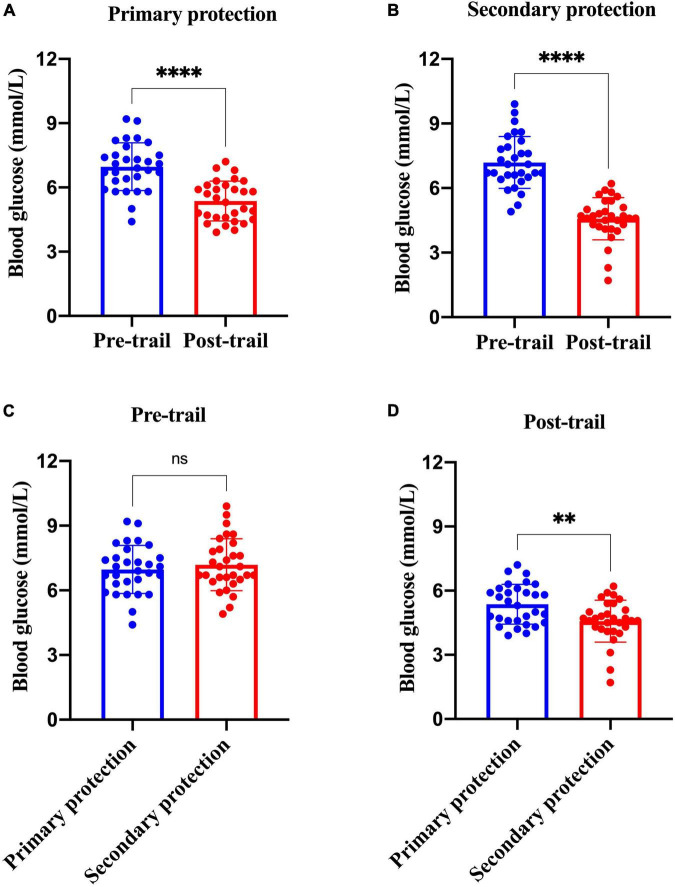
Analysis of blood glucose changes of participants with primary and secondary PPE. **(A)** The blood glucose differences of the research participants before and after the trial under the primary protection; **(B)** the blood glucose differences of the research participants before and after the trial under the secondary protection; **(C)** the blood glucose differences of under the primary protection vs. secondary protection before the trial; **(D)** the blood glucose differences of under the primary protection vs. secondary protection after the trial. ^**^p < 0.01; ^*⁣*⁣**^p < 0.0001; and ns, *p* > 0.05. PPE, personal protective equipment.

The sweat volume was roughly estimated based on weight loss before and after the trial. Although the average trial time of the participants performing moderate-intensity work under the secondary protection was 70.0 min while 180 min under the primary PPE, the average sweat volume of participants under secondary protection was 0.620 kg that was far more than the primary PPE of 0.063 kg (Supplementary Table 6). The average sweat volume of participants performing light-intensity work under secondary PPE was 0.510 kg (Supplementary Table 6). Meanwhile, the blood glucose of all participants dropped after the trial (*p* < 0.0001; Figures 7A,B). Additionally, the blood glucose and weight of the participants before the beginning of the trial under primary protection and secondary protection were similar (*p* > 0.05; Figure 7C). After the trial, the blood glucose level of the participants under secondary protection (4.57 ± 0.98 mmol/L) was lower than that of primary protection (5.36 ± 0.93 mmol/L; Figure 7D; *p* < 0.01).

### Analysis of the Physiological Index Changes of Participants Between Pre-trial and Post-trial

The differences in heart rate, blood oxygen, respiratory rate, oral/axillary temperature, and blood glucose between pre-trial and post-trial in all sub-group participants were statistically significant (*p* < 0.05; Table 4). The changes of physiological index in participants performing moderate-intensity work under secondary PPE were the most dramatic.

**TABLE 4:** Analysis of the physiological index changes of participants between pre-trial and post-trial.

Variable	Differences between pre-trial and post-trial					
	Light-intensity with secondary PPE	*P* value	Moderate-intensity with primary PPE	*P* value	Moderate-intensity with secondary PPE	*P* value
Heart rate, beats/min	40.80 ± 12.50	< 0.001^a^	21.50 ± 10.86	< 0.001^a^	56.77 ± 17.32	< 0.001^a^
Blood oxygen, %	−3.40 ± 0.70	0.004^b^	−1.13 ± 14.36	< 0.001^b^	−3.07 ± 1.41	< 0.001^b^
Systolic blood pressure, mmHg	8.70 ± 10.00	0.033^b^	3.00 ± 9.95	0.112^b^	6.37 ± 16.92	0.056^b^
Diastolic blood pressure, mmHg	1.80 ± 8.18	0.44^b^	−0.43 ± 7.24	0.243^b^	3.70 ± 12.97	0.026^b^
Respiratory rate, beats/min	8.30 ± 2.11	0.005^b^	3.70 ± 1.34	< 0.001^b^	14.20 ± 3.62	< 0.001^b^
Oral temperature, °C	1.14 ± 0.33	0.005^b^	0.83 ± 0.31	< 0.001^b^	1.57 ± 0.47	< 0.001^b^
Axillary temperature, °C	1.26 ± 0.33	0.005^b^	0.85 ± 0.40	< 0.001^b^	1.92 ± 0.67	< 0.001^b^
Blood glucose, mmol/L	−2.44 ± 1.13	< 0.001^a^	−2.39 ± 1.21	< 0.001^a^	−2.62 ± 1.35	< 0.001^a^

*^a^p values were calculated using paired T-test.*

*^b^p values were calculated using the Wilcoxon matched-pairs signed-rank test.*

## Discussion

Coronavirus disease 2019 is a respiratory infectious disease that is mainly transmitted through droplets and contact and is classified as a Class B infectious disease and managed as a Class A infectious disease in China. According to the relevant technical guidelines and expert consensus (23, 26, 29), it requires that the layout of the COVID-19 isolation ward for receiving and treating patients are divided into three areas and two channels according to work needs. The three areas are the polluted area, the potentially polluted area, and the clean area, respectively. Medical workers need to enter the contaminated area to directly contact patients to carry out various medical activities with high exposure risk, and the corresponding protection level is secondary protection, and tertiary protection is required if necessary. In potentially contaminated areas, there is no need to directly contact patients. The exposure risk is medium, and the corresponding protection level is primary protection. In this study, the research participants wore primary PPE and secondary PPE to simulate clinical activities in the COVID-19 isolation ward. With the progress of the trial, there were obvious differences in the changes of various physiological indicators when the participants with the secondary PPE compared with the primary PPE, and the differences in the incidence of adverse symptoms and the duration of the trial were statistically significant.

### The Characteristics and Dynamics of Physiological Indicators in Medical Staff Wore Primary and Secondary Personal Protective Equipment With Moderate-Intensity Activity

The summer temperature of the COVID-19 isolation ward along the south-central coast in China routinely exceeds 30°C. In this study, the indoor ambient temperature of the COVID-19 isolation ward ranged from 32 to 35°C, and the relative humidity was about 60%, and the results showed that in the case of primary PPE, the heart rate of all the participants showed a slow upward trend, but the heart rate of the 180 min trial activity did not exceed 120 beats/min. However, under the secondary PPE, the heart rate of all subjects (0.54 beats/min) was much faster at the ascending speed than that of the primary PPE (0.096 beats/min). At 40 min after the trial began, the heart rates of 66.7% (20/30) participants were higher than 120 beats/min. In addition, we found that the respiratory rate of the participants with the primary PPE was stable throughout the trial, all less than 30 times/min. However, the respiratory rate of the participants with the secondary PPE showed a faster growth rate (0.016 beats/min), which was higher than that of the primary protection (0.004 beats/min). Due to the airtight characteristics and good liquid and gas barrier capabilities of their structure of protective clothing and medical protective masks, medical staff in the isolation ward of COVID-19 are more likely to cause insufficient gas exchange. The micro-environment of the human body under PPE was affected by the environment and the body’s heat dissipation, especially for secondary PPE, which accordingly triggers corresponding neural reflexes, resulting in increased breathing frequency and accelerated breathing depth (30, 31). To guarantee the personal safety of the research participants, the trial was stopped when the participants could not persist, thus no extreme changes in physiological indicators were observed.

### The Characteristics of Subjective Symptoms in Medical Staff Who Wore Primary and Secondary Personal Protective Equipment With Moderate-Intensity Activity

We collected the subjective perception information of the participants to confirm the changes in physiological indicators. We found that the proportions of palpitations (primary PPE vs. secondary PPE: 3.3 vs. 80.0%) and anhelation (primary PPE vs. secondary PPE: 3.3 vs. 63.3%) of the research participants with the secondary protection were higher than that of the primary protection at 30 min since the trial initiation. Mengyu et al. reported that when the subjects wore medical protective clothing, with the increase in exercise intensity and time, it was difficult to maintain a stable microcirculation of thermal-moist, which can lead to a more uncomfortable the subjective perception and the stronger perception of the fatigue (32). This finding was further confirmed in our trial. However, the functions of liquid barrier, microbial barrier, and anti-particle penetration are important performances for medical disposable protective clothing. Medical staff in isolation wards wear medical PPE to block viruses and bacteria. Meanwhile, medical PPE makes the trapped sub-PPE air contain more water vapor relative to the surrounded air (33), causing the body temperature to rise, which may cause a series of subjective discomfort symptom (34–39).

### The Analysis of the Safety Work Time in COVID-19 Isolation Ward

Our study revealed that medical staff performing a moderate-intensity activity for 40 min continuously in the COVID-19 isolation ward could influence their physiology. However, medical staff working in polluted areas is hard to replenish water and energy timely due to the special environment. More importantly, when medical staff is extremely tired, quickly taking off PPE in a short period may increase the risk of exposure. Therefore, a reasonable arrangement of work intensity and rest time is particularly important for medical staff with different standards of PPE in the COVID-19 isolation ward. In this study, 30 participants were tested under primary and secondary PPE with moderate-intensity activity, respectively, and the results showed that 30 participants with primary PPE persisted for more than 3 h of trial, but the median time of participants with secondary PPE was only 70 min (the range is 40–120 min). Thus, to ensure the personal safety of medical staff in the COVID-19 isolation ward in real medical work, the continuous safe working time of wearing secondary PPE in a high-temperature and high-humidity environment should be kept within 40 min. In addition, the median time of participants with secondary PPE performing light-intensity work was 110 min (the range was 90–160 min), which was between moderate-intensity work with secondary PPE and moderate-intensity work with primary PPE. Therefore, the rational arrangement of medical activities of different intensities contributes to reducing work fatigue and prolonging safe working hours.

### The Analysis of the Sweat Volume and Energy Expenditure

To further evaluate the physical exertion of the research participants in medical activities under primary protection and secondary protection, this study monitored the subjects’ body weight and blood glucose before and after the trial, and then assessed their sweating and energy consumption during the trial. The results showed that the average weight loss of the research participants with primary protection was 0.063 ± 0.076 kg, and the wet range of cotton hand-washing clothes was about 10%. The average weight loss of the research subjects wearing secondary protection was 0.62 ± 0.202 kg, and the wet range of the cotton handwashing clothes worn by the research participants was more than 80%. It was worth noting that the test duration of all research participants under the primary protection reached 180 min, while the average trial duration of the research participants under the secondary protection was only 70.0 min. Thus, the average weight loss rates of the research participants under the primary protection and secondary protection were 21 and 504 g/h, respectively. It showed that the physical exertion and sweating of medical staff under secondary protection during moderate-intensity medical activities are much higher those under primary protection. The previous literature (28) demonstrated that when the human body sweats too much in a short period, it will cause symptoms, such as increased body temperature, dryness in the mouth, nausea, and vomiting. A large amount of sweat in the protective clothing cannot evaporate, causing the protective clothing to be damp, increasing the perception of discomfort and fatigue in the human body, and then the possibility of causing accidents to medical staff is greatly increased. Additionally, all participants of this study were tested blood for glucose tests at the beginning and end of the trial respectively. The results of the study showed that there was no statistically significant difference in blood glucose levels between the research subjects of the two groups at the beginning of the test. However, the declining of the blood glucose level of the participants under the secondary protection was greater than that of the primary protection (*p* < 0.01). Given the above results and the special environment of the isolation ward, the medical staff was unable to eat, drink, and intake sugar timely. Therefore, before entering the contaminated area work, medical staff should eat properly to prevent entering the contaminated area on an empty stomach to work, especially the staff of the night shift.

### Suggestions to Reduce the Adverse Impacts of Wearing Enhanced Personal Protective Equipment

Undoubtedly, reducing exposure time and taking longer breaks benefit medical staff’s work shifts to deal with the medical work and PPE-induced discomfort. Previously, studies demonstrated that conducting a 3:1 work-rest ratio can sharply decrease thermal strain during moderate-intensity work, especially for older employees (40, 41), and the adverse effects of PPE (i.e., thirst, exhaustion, and headache) were associated with longer work shift durations (42). Therefore, we suggest that the work shifts of healthcare workers with moderate-intensity wearing PPE should be interrupted by longer breaks and older healthcare workers try to avoid the long-time medical activity of moderate intensity, which can improve the physical and cognitive performance of medical staff and thereby reduce the risk of accidental injuries and contamination. In addition, to alleviate heat strain and discomfort of healthcare workers with PPE, we can perform heat mitigation strategies, such as pre-cooling, to reduce the indoor temperature in the COVID-19 isolation ward (43–45). These strategies were validated in other studies that could relieve physiological and perceptual responses in athletes, firefighters, and military personnel (46–48). In addition, it was reported that some wearable devices [i.e., a phase change material cooling vest and PAPR (3M^®^ Versaflo^®^ TR-300 series)] could significantly improve thermal comfort among medical staff working at COVID-19 wards wearing PPE (49, 50). Lastly, the psychological state can directly affect physiological responses (51, 52), and we suggest that clinical managers should pay attention to the mental status of medical staff, promptly identify problems, and provide guidance to affected workers, and reduce their working hours and duration of wearing PPE (53).

### The Limitations in This Study

Certain limitations of our study should be acknowledged. First, the study has a small sample size, especially for the light-intensity with a secondary PPE group, and only included medical staff at a single medical center. Second, our research is limited to the study of the physiological and anthropometric parameters cited in the methodology, but the psychological indicators, such as anxiety and tension, are not measured and discussed. Third, although we stratified the working intensity to light intensity and moderate work according to the routine medical activities, the working intensity simulated in this trial did not fully reflect the working intensity of real clinical practice. Thus, the results of this study should be carefully interpreted and validated in clinical practice. Further laboratory and environmental studies examining the physiological impact of PPE among COVID-19 would be extremely beneficial.

## Conclusion

In summary, the combination of an exacerbated workload and secondary PPE worn by COVID-19 healthcare workers increases the change in physiological indicators, and in some cases the adverse symptoms, which can affect and even suspend their medical work. Thus, taking mandatory regular breaks, arranging reasonable work intensity, and maintaining optimum temperature in the working environment accord with the principle of Bioethics of a “Job Well Done” during the COVID-19 pandemic and are beneficial to safety work in medical staff.

## Data Availability Statement

The raw data supporting the conclusions of this article will be made available by the authors, without undue reservation.

## Ethics Statement

The studies involving human participants were reviewed and approved by the Ethical Committee of Fuzhou Pulmonary Hospital. The patients/participants provided their written informed consent to participate in this study.

## Author Contributions

MZ: conception, design, and finalization of the manuscript. JZ: acquisition, analysis, and interpretation of data, and revision and finalization of the manuscript. JY: acquisition of data and draft of the manuscript. XG: perform the data analysis and figures plott. All authors read and approved the final manuscript.

## Conflict of Interest

The authors declare that the research was conducted in the absence of any commercial or financial relationships that could be construed as a potential conflict of interest.

## Publisher’s Note

All claims expressed in this article are solely those of the authors and do not necessarily represent those of their affiliated organizations, or those of the publisher, the editors and the reviewers. Any product that may be evaluated in this article, or claim that may be made by its manufacturer, is not guaranteed or endorsed by the publisher.
