# Supplementary material for: The Evaluation of Physiological Index Changes and Safety Work of Female Medical Staff With Different Medical Protection Standards in the Ward of COVID-19
Source: Front Med (Lausanne). 2022 Jun 22;9:906140. doi: 10.3389/fmed.2022.906140 (PMC9256920; doi:10.3389/fmed.2022.906140)
Supplement: Supplementary file 1 [file Data_Sheet_1.pdf]

Table S1 Baseline characteristics and physical indicators of this trial.

Variable	Moderate-intensity		Light-intensity
	Primary PPE (n = 30)	Secondary PPE (n = 30)	Secondary PPE (n =10)
Age, year	35.0 (26.5, 42.5)	35.0 (26.5, 42.5)	25.0 (22.8, 26.3)
Weight, kg	54.9 ± 5.4	54.9 ± 5.4	53.5 ± 3.6
Heart rate, beats/min	84 (78, 87)	77 (73, 82)	77 (72, 84)
Systolic blood pressure, mmHg	111.8 ± 11.2	105.9 ± 9.1	109.1 ± 14.78
Diastolic blood pressure, mmHg	72.6 ± 7.1	68.8 ± 6.1	67.8 ± 10.24
Respiratory rate, beats/min	20 (19, 20)	19 (18, 19)	18.7 ± 0.67
Oral temperature, ℃	36.6 ± 0.3	36.4 ± 0.2	36.54 ± 0.18
Axillary temperature, ℃	36.1 (35.1, 36.3)	36.0 (35.9, 36.2)	36.1 (35.6, 36.2)
Blood oxygen, %	99.0 (98.0, 99.0)	98.5 (98.0, 99.0)	99.0 (98.8, 99.0)
Blood glucose, mmol/L	7.2 ± 1.2	7.0 ± 1.1	7.3 ± 1.0

Table S2 The primary and secondary personal protective equipment (PPE) of this trial.

Primary PPE	Secondary PPE
Disposable isolation gown	Medical protective gowns
Disposable medical cap	Disposable medical cap
Surgical mask	N95 medical mask
Rubber surgical gloves	Rubber surgical gloves
Medical shoe covers	Medical goggles
	Medical shoe covers

Abbreviation: PPE, personal protective equipment.

Table S3 Medical protective equipment in this study

Name	Brand	Model and specification
Disposable medical cap	Esound Medical Device CO.,Ltd	-
N95 medical mask	Winner Medical	N95 folding
Surgical mask	Winner Medical	17cm×9cm-3P
Medical goggles	Xinghui Optics (Xiamen) Co.,Ltd	G200-C
Medical protective gowns	Winner Medical	Coverall
Disposable isolation gown	Winner Medical	130×140 cm
Rubber surgical gloves	Guilin Zizhu latex products Co.,Ltd.	-
Medical shoe covers	Henan Bochuang Medical Technology CO.,Ltd	52×42×42.5cm

Table S4 Main instruments in this study

Measured parameters	Brand and Model Names	Measuring Range	Measuring precision
Body temperature (armpit)	Jingchuang (Tlog100EH)	-30 ~ 70℃	±0.3℃ (-20 ~ 40℃)
Body temperature (mouth)	Omron (MC-246)	32 ~ 42℃	±0.1℃
Heart rate	Omron (HEM-7130)	-	±3mmHg (±0.4kPa)
Blood pressure	Omron (HEM-7130)		
Blood glucose	Abbott (FreeStyle OptiumNeo)	-	-
Oxygen saturation	Heal Force (PC-60B)	-	-
ECG	Nihon Kohden (ECG-1350P)	-	-
Bathroom scale	YUANYAN MEDICAL (RGZ-120)	0 ~ 120 kg	-

Abbreviation: ECG, Electrocardiograph.

Table S5 Analysis of incidence in subjective symptoms of participants with primary and secondary personal protective equipment (PPE).

Protection standard	Symptoms	The incidence of symptoms (%)							P [a]
		0 min	30 min	60 min	90 min	120 min	150 min	180 min	
Primary	Dry mouth	0.00	30.00	60.00	76.67	93.33	96.67	100.00	<0.001
	Dizziness	0.00	0.00	0.00	6.67	16.67	33.33	63.33	<0.001
	Palpitations	0.00	3.33	3.33	6.67	16.67	20.00	20.00	<0.001
	Anhelation	0.00	3.33	3.33	10.00	10.00	16.67	23.33	<0.001
Secondary	Dry mouth	0.00	80.00	89.29					<0.001
	Dizziness	100.00	23.33	71.43					<0.001
	Palpitations	100.00	80.00	89.29					<0.001
	Anhelation	100.00	63.33	78.57					<0.001

[a] The P values were calculated using the trend Chi-square tests.

Abbreviation: PPE, personal protective equipment.

Table S6 Comparison of the weight losses (sweat volume) between before and after the trail.

Work intensity	PPE	N	Weight loss (kg)	P [a]	P [b]	P [c]
Moderate	Primary protective	30	0.063 ± 0.076	<0.001	-	-
Moderate	Secondary protection	30	0.620 ± 0.202		-	-
Light	Secondary protection	10	0.510 ± 0.379	-	0.114	<0.001

[a] P value was calculated using paired T test for moderate-intensity with primary PPE vs. moderate-intensity with secondary PPE.

[b] The P value was calculated using independent-samples T test for light-intensity with secondary PPE vs. moderate-intensity with secondary PPE.

[c] The P value was calculated using independent-samples T test for light-intensity with secondary PPE vs. moderate-intensity with primary PPE.

Abbreviation: PPE, personal protective equipment.
